# Hyperthyroidism in the personalized medicine era: the rise of mathematical optimization

**DOI:** 10.1098/rsif.2019.0083

**Published:** 2019-06-26

**Authors:** Fanwen Meng, Enlin Li, Paul Michael Yen, Melvin Khee Shing Leow

**Affiliations:** 1 Health Services and Outcomes Research, National Healthcare Group, Singapore; 2 Cardiovascular and Metabolic Disorders Programme, Duke-NUS Medical School, Singapore; 3 Department of Endocrinology, Tan Tock Seng Hospital, Singapore; 4 Lee Kong Chian School of Medicine, Nanyang Technological University, Singapore; 5 Yong Loo Lin School of Medicine, National University of Singapore, Singapore; 6 Clinical Nutrition Research Centre, Singapore Institute for Clinical Sciences, A*STAR, Singapore; 7 Changi General Hospital, Singapore

**Keywords:** Graves' disease, anti-thyroid drug dosing, personalized medicine, ordinary differential equation modelling, mathematical optimization

## Abstract

Thyroid over-activity or hyperthyroidism constitutes a significant morbidity afflicting the world. The current medical practice of dose titration of anti-thyroid drug (ATD) treatment for hyperthyroidism is relatively archaic, being based on arbitrary and time-consuming trending of thyroid function that requires multiple clinic monitoring visits before an optimal dose is found. This prompts a re-examination into more deterministic and efficient treatment approaches in the present personalized medicine era. Our research project seeks to develop a personalized medicine model that facilitates optimal drug dosing via the titration regimen. We analysed 49 patients' data consisting of drug dosage, time period and serum free thyroxine (FT4). Ordinary differential equation modelling was applied to describe the dynamic behaviour of FT4 concentration. With each patient's data, an optimization model was developed to determine parameters of synthesis rate, decay rate and IC_50_. We derived the closed-form time- and dose-dependent solution which allowed explicit estimates of personalized predicted FT4. Our equation system involving time, drug dosage and FT4 can be solved for any variable provided the values of the other two are known. Compared against actual FT4 data within a tolerance, we demonstrated the feasibility of predicting the FT4 subsequent to any prescribed dose of ATD with favourable accuracy using the initial three to five patient-visits' data respectively. This proposed mathematical model may assist clinicians in rapid determination of optimal ATD doses within allowable prescription limits to achieve any desired FT4 within a specified treatment period to accelerate the attainment of euthyroid targets.

## 1. Introduction

Graves' disease is the most common cause of hyperthyroidism, a ubiquitous disorder that afflicts a large segment of people worldwide. Although its life-threatening complications such as thyroid crisis, thyrocardiac failure and thyrotoxic periodic paralysis are less common manifestations, Graves' disease can be disabling and result in significant healthcare costs and economic loss as many affected are in their prime of life and productivity. Every year, 0.02–0.05% of the population will be newly diagnosed with Graves' disease [1]. It is an autoimmune disorder specifically characterized by thyroid stimulating hormone (TSH) receptor autoantibodies which bind to thyroid gland TSH receptors resulting in unrestrained production of thyroid hormones [2].

Currently, there are three established treatment methods for Graves' disease, i.e. anti-thyroid drugs (ATD) to block the production of thyroid hormones or ablative strategies such as radioactive iodine ablation and total thyroidectomy followed by lifelong thyroid hormone replacement.

Multiple factors including patient preference and risk of relapse are involved in determining the choice of treatment modality for each patient [3,4]. ATDs are frequently prescribed at the outset to achieve rapid control of hyperthyroidism. The most widely used ATD belongs to the thionamide class, of which methimazole and carbimazole are more commonly used [5]. Carbimazole is metabolized to methimazole in the body. Another thionamide is propylthiouracil, which is generally less preferred except for certain clinical circumstances because of the higher risks of side effects [6].

Currently, there are two regimens that clinicians can adopt in administering ATD to patients: the titration regimen and the block-and-replace regimen [5]. The titration regimen involves adjusting the ATD dosage based on thyroid function tests (TFT) such that the patient ultimately achieves euthyroidism at the lowest dose of ATD. On the other hand, in block-and-replace regimen, the patient will be given high doses of ATD to suppress production of thyroid hormones and co-prescribed with levothyroxine replacement to avoid hypothyroidism. Both regimens have comparable effectiveness, but the titration regimen has less adverse effects to the patients [7,8]. However, the titration regimen has the drawback of more frequent clinician visits for monitoring and dose titration [8]. Besides inhibiting the production of thyroid hormones, ATDs also have immunosuppressive effects that lead to a reduction of autoantibodies [9–11]. Even though ATD has direct effects on the immune system, this is not dose-dependent and it could be that direct immunosuppressive effects are already maximized at a low dose of ATD [12]. It is also likely that the immunosuppressive effects are actually a result of the normalization of the thyroid hormones to the euthyroid state [11,12]. Therefore, it is important that patients achieve euthyroidism as soon as possible with the lowest ATD dose so as to minimize side effects and maximize the thyroid-specific immunosuppressive effects. However, in current clinical practice, titration of ATD has been an arbitrary exercise based on clinical judgement and preceding TFT results. It takes much time and frequent monitoring for clinicians to find the optimal drug dosage using such a trial-and-error strategy which increases the costs for patients. Thus far, there has been little research on optimizing the dose titration of ATDs, a situation that has persisted for decades.

Motivated by the above, we attempt to develop a mathematical model that could guide clinicians in predicting the optimal drug dosage for patients with Graves' disease to attain euthyroidism. Specifically, we apply ordinary differential equation (ODE) to describe the dynamic behaviour of free plasma thyroxine (FT4) concentration and introduce a mathematical optimization model to estimate parameters involved (i.e. synthesis rate, decay rate and IC_50_—concentration of ATD which reduces FT4 synthesis by half) in the differential equation with sets of TFT data for each patient. We derive an individualized closed-form approximation of FT4 concentration in terms of ATD dosage and time.

We conducted numerical experiments with the TFT data of 49 patients. The data were acquired in a retrospective study entitled, ‘Elucidating the dose response relationship of thyroid hormone to anti-thyroid drugs in Graves' disease', and ethically approved by the Domain Specific Review Board (DSRB reference code—C/2011/02012) of National Healthcare Group, Singapore. This study was determined by the DSRB to be an exempt category with no requirement for consent from patients in view of the retrospective nature and de-identified data. Favourable results were obtained in predicting the FT4 value in response to any ATD dose. In particular, compared against actual FT4 data within a tolerance, favourable accuracy rate (77.1%, 75.0% and 83.9%) of predicted FT4 using data from the first three, four and five visits, respectively, was achievable. The proposed model has a potential benefit for clinicians to determine optimal drug dosage for patients with Graves' disease to achieve a desired FT4 value within a pre-determined time period.

## 2. Methods

In this paper, ODE was employed to describe a mathematical relationship between the rate of change of FT4 and its output (i.e. synthesis and secretory rate) and decay rates. The thyroid hormone output rate is dependent on ATD dosage. This will result in a mathematical relationship between rate of change of FT4 concentration and ATD dosage. Integration of the ODE will then yield the explicit relationship between FT4 concentration and ATD dosage. Parameters in the equation can be obtained after fitting in a few sets of TFT individualized by patients. These parameters will thus be unique to each patient. With the equation and the unique parameters for each patient, we would be able to predict the optimal ATD dosage that is needed for each patient to reach the desired target FT4 concentrations for euthyroidism within a specified time interval. We can also apply the model to predict the FT4 level for any prescribed dose of ATD if administered over a pre-determined duration. We then derive a mathematical model to predict the optimal ATD dose using carbimazole for Graves' disease patients to reach euthyroid FT4 levels. The model under consideration is applied to determine the optimal FT4 concentration for each patient based on to a few sets of TFT data. The FT4 concentration is a decreasing function over time and will converge to a certain value when time tends to infinity for any given ATD dose under any given thyroid activity. We hereby list some notations such as parameters and variables which will be used in model development.

Variables
— *y*: free thyroxine concentration (pmol l^−1^)
— *d*: anti-thyroid drug dosage (mg)
— *t*: time (day)

*Parameters*

— *A*: synthesis rate of *y*
— *C*: constant decay rate
— IC_50_: dosage of ATD that reduces *y* by half

By definition, y>0, d≥0, t≥0, and parameters *A*, *C*, IC_50_ are all positive. The effect of the ATD dosage at *d* mg was derived by taking reference from the Hill equation [13], which is a sigmoidal curve that is commonly used to relate the drug concentration to its effect. Denote the effect of ATD at dosage *d* mg to be $\;\;d/(IC_{50}+d)$, where IC_50_ is defined here as the drug dosage that will result in half the maximum inhibitory effect on the FT4 synthesis rate. Note that this expression fulfils the following properties that describe how the effect of the drug would have varied with drug dosage *d* mg:

— when $d=IC_{50},\;\;\;\;\;\;\frac{d}{IC_{50}+d}=\frac{1}{2}$,
— when $d=0,\;\;\;\;\;\;\frac{d}{IC_{50}+d}=0$,
— when $d\to \infty ,\;\;\;\;\;\;\frac{d}{IC_{50}+d}\to 1.$

### 2.1. Differential equations for FT4 concentration

In this study, we use ODE modelling to describe the dynamic behaviour of serum FT4 concentration with the dosage of ATD over time. For the differential equation under consideration, the first-order derivative of *y* with respect to *t* is a polynomial of degree 2 with a non-zero coefficient of *y* and zero constant term. Namely,

2.1 $$\frac{dy}{dt}=A\left(1-\frac{d}{IC_{50}+d}\right)y-Cy^{2}.\;$$

This ODE essentially expresses that the rate of change of FT4 is dependent on the net difference between its rate of synthesis and its rate of decay. In this model, thyroid hormone synthesis and secretion rate is modelled as a function of FT4 concentration, since the thyroid hormone synthesis/secretion rate is dependent on TSH (or TSH receptor autoantibody level) which is itself dependent on FT4 level at any given time [14]. As to the second term in equation (2.1), we assume that the decay of thyroxine follows second-order kinetics instead of first-order kinetics. This is because thyroid hormone induces its own catabolism. As such, in a euthyroid state, the half-life of thyroxine is about 7 days, but its half-life shortens to about 3–4 days in the hyperthyroid state. Conversely, the half-life lengthens to 8–10 days in a hypothyroid state. Thus this assumption and equation (2.1) are biologically plausible and reasonable to describe the FT4 behaviour with time [14].

For simplicity in description, let $B(d)=1-d/(IC_{50}+d).$ It is clear that 0<B(d)<1. Rewrite equation (2.1) as $dy/dt-AB(d)y=-Cy^{2},$ which is actually in form of

$$\frac{dy}{dt}+P(t)y=Q(t)y^{n},\;\mathrm{where}\;\;P(t)=-AB(d),\;Q(t)=\;-C,\;n=2.$$

It is known that the above ODE is a Bernoulli equation with the order of n=2 and the general solution being in form of

$$y^{1-n}=y^{-1}=e^{-\int (-P(t))dt}\left(\int Q(t)\left(-e^{\int (-P(t))dt}\right)dt+C_{1}\right),$$

where *C*_1_ is an arbitrary constant. Solving the above equation, it gives that

$$y^{-1}=e^{-AB(d)t}\left(C\int e^{AB(d)t}dt+C_{1}\right)=e^{-AB(d)t}\left(\frac{Ce^{AB(d)t}}{AB(d)}+C_{1}\right).$$

Simplifying the above equation, we derive the following general solution of (2.1):

$$y(t)=\frac{AB(d)e^{AB(d)t}}{Ce^{AB(d)t}+AB(d)C_{1}}.$$

Hence, we obtain the closed-form general solution of the ODE (2.1) as below.

2.2 $$y(t)=\frac{A(1-d/(IC_{50}+d))e^{A(1-d/(IC_{50}+d))t}}{Ce^{A(1-d/(IC_{50}+d))t\;}+\;A(1-d/(IC_{50}+d))\;C_{1}\;}.$$

There are four unknown parameters in the solution function (2.2), that is, *A*, *C*, IC_50_ and *C*_1_. Evidently, *A*, *C*, IC_50_ are positive by nature. The solution function *y*(*t*) is always positive and would be decreasing over time with the ATD drug treatment. To ensure that *y*(*t*) is positive according to (2.2), we must have

$$Ce^{A(1-d/(IC_{50}+d))t}+A\left(1-\frac{d}{IC_{50}+d}\right)C_{1}>0.\;$$

After applying some mathematical manipulations, the first-order derivative of *y* with respect to t is equal to

$$y^{\prime }(t)=\frac{dy(t)}{dt}=\frac{C_{1}A^{3}B{\left(d\right)}^{3}e^{AB(d)t}}{{\left(AB(d)C_{1}+Ce^{AB(d)t}\right)}^{2}}.$$

It is known that *y*(*t*) is decreasing over time *t* if and only if $y^{\prime }(t)<0$, which is equivalent to *C*_1_ < 0. Thus, to ensure the underlying solution function *y*(*t*) to be well defined, the parameters under consideration must satisfy the following conditions:

2.3 $$Ce^{A(1-d/(IC_{50}+d))t\;}+\;A\left(1-\frac{d}{IC_{50}+d}\right)\;C_{1}>0,\;C_{1}<0.$$

The above ODE model describes the possible behaviours of FT4 concentration *in vivo*. Note that $\underset{t\to \infty }{\lim}y(t)=A\times IC_{50}/C(IC_{50}+d).$ This means that the solution function *y*(*t*) is monotonously decreasing and tends to an asymptotic constant of $A\times IC_{50}/C(IC_{50}+d)$ as time *t* tends to infinity.

### 2.2. Mathematical optimization model

The FT4 solution function *y*(*t*) under consideration can be treated as a parametric function in $A,C,IC_{50},C_{1}.$ The values of these parameters could be estimated based on the individualized TFT data. In this study, we apply a mathematical optimization approach to determine the values of the parameters of interest with individualized patient data. Consequently, we then derive the personalized FT4 function *y* in an explicit form in terms of the dosage *d* and time *t*, which may assist clinicians in medical decision-making, such as the dosage choice of ATD drug and the time interval to achieve a desired FT4 target.

In our analysis, the TFT data for each patient were paired with clinically relevant data that comprise review time interval (equivalent to each treatment duration per dose adjustment) *t*, the ATD dose *d* and FT4 value *y*. At the initial (first) review, the dose *d* = 0. Based on the tested FT4 value, the medical doctor would initiate ATD of a certain dosage and review the patient's FT4 again after a certain time period. At the next review, depending on the latest FT4 value, the doctor will adjust the drug dosage accordingly to target FT4 into the normal range. For convenience in description, the underlying data are arranged as a collection of three-dimensional vectors $(d_{i-1},t_{i},y_{i}),i=1,2,\ldots ,m$, where *i* denotes the *i*th review and *m* denotes the total number of patient visits. For $i=1,d_{0}=0,t_{1}=0,$ *y*_1_ denotes the initial FT4 value. For i=2,…,m, $d_{i-1}$ denotes the ATD dosage prescribed at the preceding review (i.e. (i−1)-th review), *t_i_* denotes the length of time period from the initial review to the *i*th review, *y_i_* denotes the tested FT4 value at the *i*th review. Then, the time interval between two consecutive reviews is equal to $t_{i}-t_{i-1}$ for i=2,…,m.

Optimization as a powerful decision-making modelling strategy has been well developed and widely applied in decision-making in management science, industrial engineering and medical decision-making [15–17]. A typical optimization model comprises three components, i.e. decision variables, objective function and constraints. Employing an optimization approach, one can find the optimal solution of decision variables from a set of alternatives (defined by the constraints) under certain decision criterion (i.e. objective function). For i=1,2,…,m, define function *f_i_* by

2.4 $$f_{i}(A,IC_{50},C,C_{1},d,t):=f(A,IC_{50},C,C_{1},d_{i-1},t_{i}),$$

where *f* is in the form of the solution function (2.2) of the ODE model. That is,

$$f(A,IC_{50},C,C_{1},d,t)=\frac{A(1-d/(IC_{50}+d))e^{A(1-d/(IC_{50}+d))t}}{Ce^{A(1-d/(IC_{50}+d))t\;}+\;A(1-d/(IC_{50}+d))\;C_{1}\;}.$$

In this study, the values of $A,C,IC_{50},C_{1}$ are determined such that each predicted FT4 value *f_i_* at $\left(d_{i-1,}t_{i}\right)$ could approximate the actual *y_i_* as close as possible, i=1,2,…,m. Along this direction, we turn to find the values of these parameters to minimize the average deviations between the predicted value of *f_i_* and the actual value of *y_i_*. In mathematics, this decision criterion can be re-formulated as the average of squared differences of *f_i_* and *y_i_* over the underlying visits. In addition to the conditions mentioned above, these four parameters are required to satisfy the initial condition of the ODE, $f_{1}(A,\;IC_{50},C,C_{1},0,0)=y_{1}$. We also impose an upper bound of 150 mg on IC_50_ according to the prescription limits of accepted clinical practice and guidelines.

The optimization model corresponding to differential equation (2.1) is as follows:

2.5 $$\begin{array}{rl} & \underset{A,C,IC_{50},C_{1}}{\min}\\ & \frac{1}{m-1}\overset{m}{\underset{i=2}{\sum }}{\left(\frac{A\times IC_{50}/(IC_{50}+{\tilde{d}}_{i})e^{\left(A\times IC_{50}\times t_{i}/IC_{50}+{\tilde{d}}_{i}\right)}}{C\times e^{A\times IC_{50}\times t_{i}/(IC_{50}+{\tilde{d}}_{i})}+\;A\times IC_{50}\times C_{1}/(IC_{50}+{\tilde{d}}_{i})}-y_{i}\right)}^{2}\\ & \mathrm{subject}\;\;\mathrm{to}\;\;\;\;\;\;A-\;y_{1}(C+A\times C_{1})=0,\;\;\;\;\;(\mathrm{initial}\;\;\mathrm{condition})\\ & Ce^{\left(A\times IC_{50}\times t_{i}/IC_{50}+{\tilde{d}}_{i}\right)}+\frac{A\times IC_{50}\times C_{1}}{IC_{50}+{\tilde{d}}_{i}}>0,\;i=1,\ldots ,m,\\ & A>0,\;C>0,\;C_{1}<0,\;0<IC_{50}\leq 150, \end{array}$$

where ${\tilde{d}}_{i}=d_{i-1},i=1,\ldots ,m.$ To aid computational implementation, we rewrite the inequality constraints of model (2.5) as below. For i=1,…,m,

$$(IC_{50}+{\tilde{d}}_{i})\ln(A\times IC_{50}\times (-C_{1}))-\;t_{i}\times A\times IC_{50}-(IC_{50}+{\tilde{d}}_{i})\ln(C(IC_{50}+{\tilde{d}}_{i}))<0.$$

Model (2.5) is a nonlinear optimization problem with a nonlinear objective function constrained by one nonlinear equality and *m* nonlinear inequalities together with some boundedness requirements. This optimization problem can be solved efficiently by using well-developed software, such as Gurobi Optimizer (www.gurobi.com) and Matlab built-in solver *fmincon* (https://au.mathworks.com/help/optim/ug/fmincon.html).

### 2.3. What-if analysis on anti-thyroid drug dosage

The estimated parameters $A,C,IC_{50},C_{1}$ are considered to be acceptable if the average predicted FT4 value could meet a pre-designated tolerance, say within a tolerance of 4.5 pmol l^−1^. Under this circumstance, the resultant FT4 concentration formula will be employed to describe the patient FT4 concentration behaviour in subsequent analysis on optimal drug dosage, suitable review interval and so on. On the other hand, for the cases who could not meet the given estimation tolerance, the proposed framework would be inappropriate to explore issues of interest like optimal drug dosage. We would suggest further monitoring and investigation on these patients instead. As we shall see in numerical *in silico* experiments, a large portion (near 80%) of patients under investigation would meet the tolerance requirement.

Denoted by $\tilde{y}(t,d)$, the FT4 concentration formula was derived above. Consider the following equation:

2.6 $$\begin{array}{rl} F(t,d,y) & :=y-\;\tilde{y}(t,d)\\ \;\;=\; & y-\frac{A(1-d/(IC_{50}+d))e^{A(1-d/(IC_{50}+d))t}}{Ce^{A(1-d/(IC_{50}+d))t\;}+\;A(1-d/(IC_{50}+d))\;C_{1}\;}=0. \end{array}$$

Here, *t* denotes the length of time period since the initial review, *d* denotes the ATD drug dosage and *y* denotes the FT4 value. As to equation (2.6), mathematically we can find the value of the third variable, given values of any two variables of *t, d, y*. A variety of efficient algorithms such as the bisection method can be used to solve equation (2.6). In this study, we are interested in addressing some basic and important questions from the clinical perspective using what-if analysis. Specifically, (i) for a given drug dosage *d*, what is the predicted value *y* of FT4 in a specified time period? (ii) For a target value *y* of FT4, what is the drug dosage *d* so as to reach this desired value in certain time? (iii) For a desired target value *y* and a given drug dosage *d*, how long will the time *t* take so as to reach the value *y*? We elaborate these issues by presenting illustrative numerical examples in the next section.

## 3. Results

We analysed 49 patients having different sets of TFT data consisting of drug dosage, time period between two consecutive visits and serum FT4. Patients had various review episodes ranging from two to 24 times. Basic descriptive statistics are shown in table 1.

**Table 1.** Descriptive statistics of thyroid function test data of 49 patients, including mean, standard deviation, median, minimum and maximum of patient review visits, serum free thyroxine and review interval.

item	review visit (time)	FT4 value (pmol l^−1^)	review interval (day)
mean	6.9	20.7	73.5
standard deviation	4.7	16.0	33.9
median	5	15	70
minimum	2	1	5
maximum	24	91	210

### 3.1. Parameter estimates

The personalized medicine model under consideration is different from classical prediction models based on regression analysis using a large set of observation data. In this study, we aim to derive an individualized FT4 approximation function predicted by a few early patient visit data. The data points associated with each patient are sequential in terms of visit times and review intervals, which were of high variability either for each individual patient or for all patients. Due to this unique feature, we used different patient datasets in analysis and derived the corresponding FT4 approximation functions, then compared estimation accuracy rates using the actual FT4 values of the data. Specifically, for an individual patient, we estimated values of parameters $A,C,IC_{50},C_{1}$ by solving optimization model (2.5) using the patient data from the first three visits, four visits and five visits of ATD drug dosage, the time period from the initial visit and the tested FT4 value at each visit, respectively. We adopted an estimation tolerance of 4.5 pmol l^−1^ to determine acceptable estimated parameters in comparison with actual FT4 values in the data. The desired estimated parameters were then used to establish the corresponding individualized FT4 concentration formula based on the derived closed-form solution functions of the ODE previously. When solving the underlying optimization model, we recorded only the solutions satisfying the convergence criteria set by the optimization solver. In this study, patients experienced different review visit episodes ranging from two to 24 review visits with mean of seven visits and median of five visits. Then, we chose different datasets for estimating the parameters, i.e. patients with the first *x*-visits and with at most the first *x*-visits if any, *x* = 3, 4, 5. Table 2 showed the performances of the proposed model concerning these different visit data together with the estimation accuracy rates.

**Table 2.** Comparison of estimation accuracy rates with different datasets.

dataset	number of patients	number of patients meeting the tolerance	estimation accuracy rate
first 3 visits	48	37	77.1%
at most first 3 visits	49	38	77.6%
first 4 visits	36	27	75.0%
at most first 4 visits	49	36	73.5%
first 5 visits	31	26	83.9%
at most first 5 visits	49	35	71.4%

As shown in table 2, the estimation prediction accuracy rate is the highest (83.9%) based on the first five visits data while it is the lowest (75.0%) with the first four visits data and the rate of 77.1% was achieved with the first three visits data. For patients with at most three visits (four visits or five visits) data, prediction accuracy rates are at least 71.4%. In addition, as we can see from table 2, the sample sizes with different datasets varied largely. For example, the patient sample size with five visits data (i.e. 31) is over 35% less than those with three visits data (i.e. 48). The corresponding prediction accuracy rates, i.e. 83.9% and 77.1%, were both favourable. Their difference (i.e. 6.8%) was acceptable somehow as the latter case handled much more patients than the former in prediction. Interestingly, table 2 showed that the obtained estimation accuracy rate using the first three visits data was slightly higher than that derived using the first four visits data. This might be due to the specific structure of the underlying personalized model—a constrained model where the number of constraints of interest varies with the number of data points used, resulting in a possible dynamic feasible set of the parameters, which may subsequently lead to uncertain performance in prediction to some extent. Another possible reason might be the high variability of patient visit times and review intervals, and the arbitrary ATD dosages in the treatment due to the heterogeneity of patients.

### 3.2. Optimal drug dosage

As an illustrative example, table 3 reported parameter estimations of several patients (i.e. patients with series numbers (S/N) 2, 16, 23) using the model (2.5) with at most first five visits patient data. We then derived the individualized FT4 concentration formulae by equation (2.2), which was shown in the last column of the table. These closed-form FT4 approximations provide a quick and easy way to predict FT4 and estimate optimal drug dosage of an individual patient of interest as well.

**Table 3.** Parameters of patients 2, 16 and 23 derived by the optimization model (2.5) with at most data from the first five visits and closed-form FT4 estimations based on formula (2.2).

patient S/N	visit times	*A*	IC_50_	*C*	*C_1_*	FT4 formula
2	9	0.052	58.151	0.003	−0.051	$\tilde{y}(t,d)=\frac{3.024/(58.151+d)e^{3.024t/(58.151+d)}}{0.003e^{3.024t/(58.151+d)}-0.154/(58.151+d)}$
16	3	5.194	72.901	0.371	−0.001	$\tilde{y}(t,d)=\frac{378.648/(72.901+d)e^{378.648t/(72.901+d)}}{0.371e^{378.648t/(72.901+d)}-0.379/(72.901+d)}$
23	4	4.237	35.062	0.246	−0.033	$\tilde{y}(t,d)=\frac{148.558/(35.062+d)e^{148.558t/(35.062+d)}}{0.246e^{148.558t/(35.062+d)}-4.902/(35.062+d)}$

We compared actual FT4 values and predicted values using the estimated FT4 formulae. The results were demonstrated in table 4. We can see that the overall predicted FT4 values were close to the actual values from patient TFT data. We also noted that there was a relatively big FT4 difference at the third review visit for patient 2, compared to the actual one.

**Table 4.** Comparisons of actual and predicted FT4 values of patients 2, 16, 23 using FT4 formulae shown in table 3 derived with at most data from the first five visits.

patient S/N	visits	ATD dosage (mg)	review interval (day)	actual FT4 (pmol l^−1^)	predicted FT4 (pmol l^−1^)
2	1st	0	0	75	—
2	2nd	30	77	13	11.9
2	3rd	15	28	7	13.9
2	4th	10	168	16	14.8
2	5th	5	168	15	16.0
2	6th	5	91	15	16.0
2	7th	0	98	25	17.3
2	8th	5	84	13	16.0
2	9th	5	203	13	16.0
16	1st	0	0	14	—
16	2nd	15	90	13	11.6
16	3rd	17.5	78	10	11.3
23	1st	0	0	40	—
23	2nd	15	35	16	12.1
23	3rd	15	84	10	12.1
23	4th	7.5	84	13	14.2

Using what-if analysis and the derived FT4 formula $\tilde{y}$, we estimated FT4 values under various scenarios of time *t* and drug dosage *d*. For any given target FT4 value and certain time period, the optimal drug dosage can be derived to achieve the desired FT4 value by solving the associated equation (2.6). To illustrate the above issues, we can use patient 2 as an example. The initial serum FT4 was 75 pmol l^−1^ and the estimated FT4 formula $\tilde{y}(t,d)$ is shown in table 3.

We report serum FT4 predictions and optimal drug dosages under different scenarios, respectively, in tables 5 and 6. As shown in table 5, the lower the dose, the higher the FT4, while the higher the dose, the lower the FT4. Also, for any given dose, the longer the treatment interval period, the lower the FT4 level. In practice, it is tedious to calculate the exponential terms involved in equation (2.6). We may simplify these terms using Taylor expansion and establish a simpler approximation involving polynomial terms, which would be easier for clinicians to estimate FT4 values. Using terms up to the second order in the Taylor expansion, we can derive the FT4 estimation, denoted by $\tilde{y}$, in the following way:

$$\tilde{y}=\frac{1}{z},\;\mathrm{where}\;z=C_{1}+\frac{C(IC_{50}+d)}{A\times IC_{50}}-AC_{1}\left(1-\frac{d}{IC_{50}+d}\right)t+\;\frac{1}{2}C_{1}A^{2}{\left(1-\frac{d}{IC_{50}+d}\right)}^{2}t^{2}.$$

**Table 5.** FT4 estimates of patient 2 by the model with data from the first five visits for any given dosages and review periods with an initial FT4 value of 75 pmol l^−1^.

dose (mg)	time period (day)	predicted FT4 (pmol l^−1^)
3	35	19.4
	56	17.4
	63	17.1
5	35	18.8
	56	16.9
	63	16.6
8	35	18.1
	56	16.2
	63	15.9
10	35	17.6
	56	15.8
	63	15.5
13	35	16.9
	56	15.2
	63	14.9

**Table 6.** Optimal dose estimates of patient 2 by the model with data from the first five visits for targeted FT4 values and review time periods with an initial FT4 value of 75 pmol l^−1^.

target FT4 value (pmol l^−1^)	time period (day)	dose (mg)
12	35	44.1
	42	39.9
	49	36.6
	56	34.0
	63	32.1
15	35	22.3
	42	18.3
	49	15.4
	56	13.4
	63	12.0
17	35	12.7
	42	8.9
	49	6.3
	56	4.6
	63	3.5

Via a computerized algorithm or software app, medical doctors can readily estimate FT4 values using the above formula by entering the quantities of review time *t* and drug dosage *d* of interest. According to table 6, patients may need to take higher drug doses to achieve a lower desired FT4 within the same time period. Using the model, it is also possible to determine a reasonable time frame to achieve a feasible FT4 target if the maximum carbimazole dose constraint of 60 mg is applied. For instance, if the targeted FT4 is 10 pmol l^−1^, by solving equation (2.6), we derive that the patient would need at least 42 days (i.e. six weeks) to achieve the target with the daily dosage of 60 mg.

**Figure 1.**
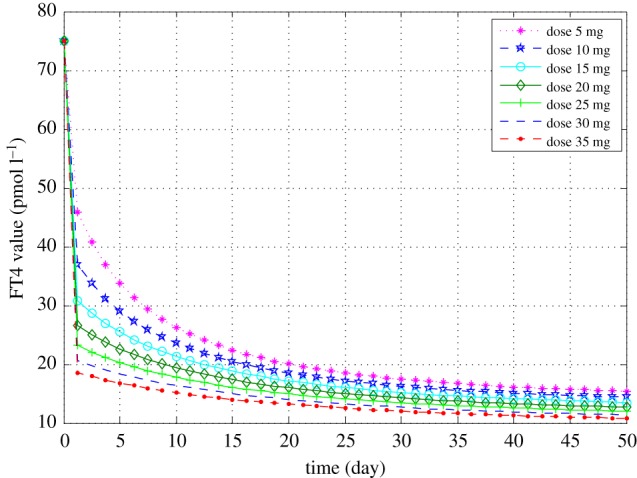
Predicted FT4 curves of patient 2 based on the estimations derived by the model using data from the first five visits for different drug dosages with an initial FT4 value of 75 pmol l^−1^. (Online version in colour.)

**Figure 2.**
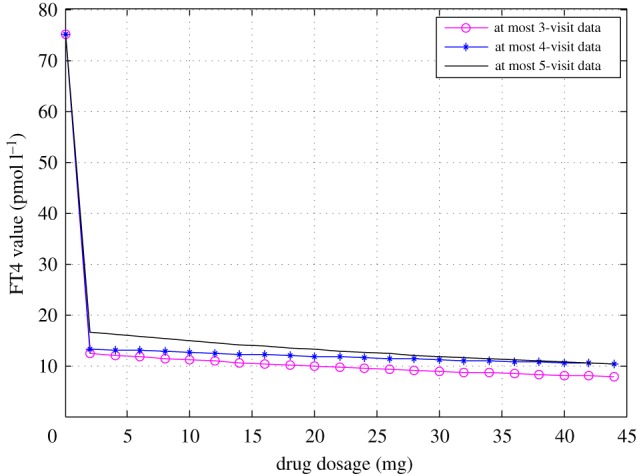
Predicted FT4 curves of patient 2 in 42 days derived by the model with three datasets with an initial FT4 value of 75 pmol l^−1^. (Online version in colour.)

Figure 1 demonstrated predicted FT4 normalization in 50 days with different doses. The higher the dose, the steeper the FT4 curve in the decline phase. Patients with the lower drug dose would take a longer time to achieve the same FT4 target. These results are in alignment with clinical treatment in practice as expected. We also investigated the predicted FT4 curves based on different visit data. As shown in figure 2, patients may take lower drug doses to achieve the same desired FT4 target when the FT4 approximation was based on first fewer visit data.

**Figure 3.**
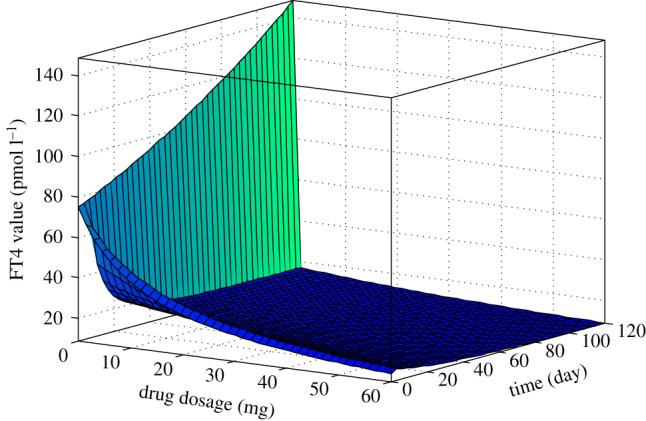
FT4 curve of patient 2 in terms of drug dose and time period rendered as a three-dimensional surface plot based on the formula derived by the model with data from the first five visits with an initial FT4 value of 75 pmol l^−1^. (Online version in colour.)

Figure 3 demonstrates the graph of the FT4 curve in terms of time and ATD drug dosage of patient 2. The graph shows that the FT4 value declines with the increases of time and drug dose. We can also see that when the dosage equals zero, the FT4 value exponentially increases with time, a situation that would be expected for patients who refuse or default ATD treatment. On the other hand, at time *t* = 0, the FT4 value decreases when ATD dose *d* increases.

## 4. Discussion

In the quest to pioneer, a personalized medicine strategy to manage hyperthyroid Graves' disease patients using ATDs, mathematical modelling based on ODE was employed to derive the solution for which we then applied our optimization strategy to the solution. We tested the ability of the model to predict FT4 values given specific prescribed doses administered over specified durations. The results are very promising as we showed that the predicted FT4 levels for any given ATD dose and time were quite close to the actual FT4 values.

This study introduced an optimization approach to estimate the underlying parameters. The optimization model is different from the least square method which has been widely used to estimate the parameters in regression models. Specifically, the proposed optimization method is a constrained model which takes into account some considerations from a practical perspective, such as the initial condition, the range of IC_50_, and the trend of FT4 as a function of time. The underlying method is a personalized model in that we estimate the individualized numerical values of the parameters using each patient's data. This contrasts with statistical modelling via the method of least squares in regression analysis which is an unconstrained model that relies on a large set of pairs of observations from patients such that the dataset for parameter fitting is separate from the validation dataset to avoid yielding overoptimistic results [18,19].

Very often, physicians would start with a dose that was heuristically estimated to be sufficiently high to suppress thyroid hormone synthesis and secretion upon diagnosis of hyperthyroidism due Graves' disease. Depending on the appropriateness of the chosen dose and duration of treatment, the patient will be targeted for clinical review with the expectation that the subsequent FT4 level would fall within the normal range. But this may not always be achieved optimally and the patient may thus take quite a protracted period prior to the plasma or serum FT4 result achieving levels within the normal population limits.

Here, we investigated the feasibility of a novel treatment paradigm according to an individualized mathematical model that allows the clinician to select the appropriate dose of ATD (e.g. carbimazole) that will predictably alter circulating FT4 to a desired level over a pre-determined duration. For instance, a common clinical scenario would be solving for a safe dose of carbimazole to be prescribed with the aim of lowering an initial serum FT4 of 50 pmol l^−1^ upon diagnosis of thyrotoxicosis from Graves' disease to a pre-specified target FT4 level of 20 pmol l^−1^ to be attained over eight weeks using our mathematical modelling approach. Also, we wish to elucidate the feasibility of discovering the optimal dose that would achieve a desired target FT4 level within a duration specified by the doctor to suit a certain time frame corresponding to the follow-up interval. We developed a mathematical model to achieve the above objectives and illustrated how the solutions are unique to each patient so that ATD dosing is individualized to euthyroid targets.

### 4.1. Limitations

This entire dissertation is focused on FT4 targets with no attempt made to find the time to achieve normalization of TSH. In hyperthyroidism, TSH is often suppressed and remains undetectable due to the phenomenon of hysteresis [20,21]. However, the present endeavour is concerned only with normalization of FT4 because the threat to life is directly dependent on the degree of elevation of FT4 beyond the normal ‘euthyroid' range. TSH, being a reflection of the response of the hypothalamus–pituitary–thyroid axis to FT4, is comparatively less critical for normalization from the perspective of risk reduction of mortality or other serious sequelae from hyperthyroidism. Obviously, the normalization of FT4 itself is not to be misconstrued as having achieved an ‘euthyroid’ state *per se* as various tissues of the body recover from hyperthyroid state at different rates [22,23]. For the latter, it is important to understand that each individual has a unique euthyroid set point which may be determined after the TSH hysteretic suppression has resolved [14,20,21,24–26]. Nevertheless, the achievement of normal circulating FT4 levels is a target of clinical priority taking precedence over the normalization of TSH especially when hyperthyroid patients need to be stabilized quickly in order to optimize the patient's health status for various reasons (e.g. surgery, thyrocardiac disease, myocardial ischemia, rapid atrial fibrillation). Yet another limitation is that any unique solution curve of an individual might not necessarily imply that the same solution holds true for the exact same patient who relapses with plasma FT4 of different level compared with the initial FT4 at first diagnosis of hyperthyroidism, since the effect of ageing on the responsiveness of the hypothalamic–pituitary–thyroid axis is not taken into account in this modelling exercise. A clinical study might be undertaken in future to see if it is possible to accurately predict FT4 given any ATD dose chosen to treat a relapse based on a known curve of the same patient using parameters derived from a previous solution curve for that patient.

## 5. Conclusion

The current practice of titration of ATD for treating Graves' disease based on TFT trend is empirical and time-consuming before an optimal dose is found. In this age of artificial intelligence and precision medicine, antiquated medical practices as such should be superseded by modern approaches that promulgates efficiency, accuracy and cost-effectiveness while eradicating guesswork from the clinicians' modus operandi as much as possible. Our personalized medicine model permits optimal drug dosing of ATD based on parameters such as synthesis rate, decay rate and IC_50_. We developed an equation system involving three variables, i.e. time, drug dosage and FT4, for solving the value of any variable provided the values of the other two are known. Compared against actual FT4 data within a tolerance of 4.5 pmol l^−1^, favourable accuracy was attainable. The proposed mathematical model when integrated into computerized algorithms and even mobile phone apps in the near future may assist clinicians in rapidly determining optimal ATD dosages to achieve any desired serum FT4 value within any specified treatment period to accelerate achievement of euthyroid targets.

## Supplementary Material

Copy of patient data.xlsx

## Supplementary Material

FT4_3Dsurface.m

## Supplementary Material

FT4andDose_calculation.m

## Supplementary Material

FT4cures_different_dataset.m

## Supplementary Material

FT4curves_different_dose.m

## Supplementary Material

main.m

## Supplementary Material

objfun.m

## Supplementary Material

constraint.m

## Data accessibility

Data used in this work are available and uploaded as electronic supplementary material together with the Matlab codes.

## References

[1] Smith TJ, Hegedus L 2016 Graves' disease. N. Engl. J. Med. 375, 1552–1565. (10.1056/NEJMra1510030) 27797318

[2] Weetman AP 2000 Graves’ disease. N. Engl. J. Med. 343, 1236–1248. (10.1056/NEJM200010263431707) 11071676

[3] Bahn RS et al. 2011 Hyperthyroidism and other causes of thyrotoxicosis: management guidelines of the American thyroid association and American association of clinical endocrinologists. Thyroid 21, 593–646. (10.1089/thy.2010.0417) 21510801

[4] Doi SAR, Loutfi I, Al-Shoumer KAS 2001 A mathematical model of optimized radioiodine-131 therapy of Graves' hyperthyroidism. BMC Nucl. Med. 1, 1 (10.1186/1471-2385-1-1) 11570980 PMC56607

[5] Bahn RS 2015 Graves’ disease: a comprehensive guide for clinicians. New York, NY: Springer.

[6] Franklyn J 2009 Antithyroid therapy—best choice of drug and dose. Nat. Rev. Endocrinol. 5, 592–594. (10.1038/nrendo.2009.201) 19844246

[7] Abraham P, Avenell A, McGeoch SC, Clark LF, Bevan JS 2010 Antithyroid drug regimen for treating Graves' hyperthyroidism. Cochrane Database Syst. Rev., 1, CD003420. 10.1002/14651858.CD003420.pub4 PMC6599817 20091544

[8] Vaidya B, Wright A, Shuttleworth J, Donohoe M, Warren E, Brooke A, Gericke CA, Ukoumunne OC 2014 Block and replace regime versus titration regime of antithyroid drugs for the treatment of Graves’ disease: a retrospective observational study. Clin. Endocrinol. 81, 610–613. (10.1111/cen.12478) 24801484

[9] Dalan R, Leow MK 2012 Immune manipulation for Graves' disease: re-exploring an unfulfilled promise with modern translational research. Eur. J. Intern. Med. 23, 682–691. (10.1016/j.ejim.2012.07.007) 22877994

[10] Ratanachaiyavong S, McGregor AM 1985 Immunosuppressive effects of antithyroid drugs. Clin. Endocrinol. Metab. 14, 449–466. (10.1016/S0300-595X(85)80042-6) 2415278

[11] Volpe R 1994 Evidence that the immunosuppressive effects of antithyroid drugs are mediated through actions on the thyroid cell, modulating thyrocyte–immunocyte signaling: a review. Thyroid 4, 217–223. (10.1089/thy.1994.4.217) 7522684

[12] Laurberg P 2006 Remission of Graves’ disease during anti-thyroid drug therapy. Time to reconsider the mechanism? Eur. J. Endocrinol. 155, 783–786. (10.1530/eje.1.02295) 17132745

[13] Gesztelyi R, Zsuga J, Kemeny-Beke A, Varga B, Juhasz B, Tosaki A 2012 The Hill equation and the origin of quantitative pharmacology. Arch. His. Exact Sci. 66, 427–438. (10.1007/s00407-012-0098-5)

[14] Goede SL, Leow MK 2018 Thyroid systems engineering: a primer in mathematical modeling of the hypothalamus–pituitary–thyroid axis. Delft, Netherlands: River Publishers.

[15] Boyd S, Vandenberghe L 2004 Convex optimization. Cambridge, UK: Cambridge University Press.

[16] Capan M et al. 2017 From data to improved decision: operations research in healthcare delivery. Med. Decis. Making 37, 849–859. (10.1177/0272989X17705636) 28423982

[17] Denton B, Kurt M, Shah N, Bryant S, Smith S 2009 Optimizing the start time of statin therapy for patients with diabetes. Med. Decis. Making 29, 351–367. (10.1177/0272989X08329462) 19429836

[18] Bates DM, Watts DG 1988 Nonlinear regression analysis and its applications. New York, NY: Wiley.

[19] Moore D, McCabe G 2003 Introduction to the practice of statistics. London, UK: W. H. Freeman.

[20] Goede SL, Leow MK 2013 General error analysis in the relationship between free thyroxine and thyrotropin and its clinical relevance. Comput. Math. Methods Med. 2013, 831275 (10.1155/2013/831275) 24082916 PMC3780511

[21] Leow MK 2016 A review of the phenomenon of hysteresis in the hypothalamus–pituitary–thyroid axis. Front. Endocrinol. 7, 64 (10.3389/fendo.2016.00064) PMC4905968 27379016

[22] Ohba K, Leow MK, Sinha RA, Lesmana R, Liao XH, Ghosh S, Refetoff S, Sng JC, Yen PM 2016 Desensitization and incomplete recovery of hepatic target genes after chronic thyroid hormone treatment and withdrawal in male adult mice. Endocrinology 157, 1660–1672. (10.1210/en.2015-1848) 26866609 PMC4816733

[23] Ohba K et al. 2017 Changes in hepatic TR*β* protein expression, lipogenic gene expression, and long-chain acylcarnitine levels during chronic hyperthyroidism and triiodothyronine withdrawal in a mouse model. Thyroid 27, 852–860. (10.1089/thy.2016.0456) 28457184 PMC5467114

[24] Goede SL, Leow MK, Smit JW, Dietrich JW 2014 A novel minimal mathematical model of the hypothalamus–pituitary–thyroid axis validated for individualized clinical applications. Math. Biosci. 249, 1–7. (10.1016/j.mbs.2014.01.001) 24480737

[25] Goede SL, Leow MK, Smit JW, Klein HH, Dietrich JW 2014 Hypothalamus–pituitary–thyroid feedback control: implications of mathematical modeling and consequences for thyrotropin (TSH) and free thyroxine (FT4) reference ranges. Bull. Math. Biol. 76, 1270–1287. (10.1007/s11538-014-9955-5) 24789568

[26] Leow MK, Goede SL 2014 The homeostatic set point of the hypothalamus–pituitary–thyroid axis—maximum curvature theory for personalized euthyroid targets. Theor. Biol. Med. Model. 11, 35 (10.1186/1742-4682-11-35) 25102854 PMC4237899

